# A Comparison of the Prevalence of the Metabolic Syndrome among Sri Lankan Patients with Type 2 Diabetes Mellitus Using WHO, NCEP-ATP III, and IDF Definitions

**DOI:** 10.1155/2018/7813537

**Published:** 2018-08-07

**Authors:** H. M. M. Herath, N. P. Weerasinghe, T. P. Weerarathna, A. Amarathunga

**Affiliations:** ^1^Department of Medicine, Faculty of Medicine, University of Ruhuna and University Unit, Teaching Hospital, Karapitiya, Galle, Sri Lanka; ^2^Department of Microbiology, Faculty of Medicine, University of Ruhuna, Galle, Sri Lanka; ^3^Department of Medicine, Faculty of Medicine, University of Ruhuna, Galle, Sri Lanka

## Abstract

**Background:**

Presence of metabolic syndrome (MetS) in patients with type 2 diabetes mellitus (type 2 DM) increases the risk of cardiovascular morbidity and mortality. Therefore, recognition of MetS in type 2 DM is important in initiating the appropriate preventive and therapeutic measures. The commonly used definitions have similarities and discrepancies. Aims of this study was to investigate the prevalence of MetS among patients with type 2DM using all three well known (WHO, IDF, and NCEP-ATP III) definitions and to identify the concordance and the difference of these three definitions.

**Methods:**

This cross-sectional study included patients with type 2 DM who were followed up at the regional diabetes centre in Galle, Sri Lanka. A total of 2913 type 2 DM patients were recruited by convenient sampling method, and their clinical and biochemical data were collected.

**Results:**

The mean age (SD) of the sample was 49.9 (10.2) years and the mean duration of diabetes was 5.04 (5.71). Prevalence of MetS was highest by WHO (70%) followed by IDF (44%) and NCEP-ATP III (29%) definitions. The prevalence was significantly higher in women according to all three definitions, and the difference was most marked with NCEP-ATP III and IDF definitions. Around 25% were identified as having MetS by all three definitions whereas around 45% were recognized with MetS by two definitions. While concordances between WHO with IDF (0.37,* p* < 0.001) and NCEP-ATP III (0.24,* p* < 0.001) criteria were poor, they were average (0.53,* p* < 0.001) between NCEP-ATP III and IDF criteria.

**Conclusions:**

The prevalence of MetS among patients with type 2 DM can significantly be varied based on the definition used and the three definitions of MetS recognized different set of individuals. The highest prevalence of MetS was observed with WHO (70.6%) whereas lowest was observed with NCEP-ATP III definition.

## 1. Introduction

Type 2 diabetes mellitus (type 2 DM) has become a global epidemic with significant disability, premature death, and enormous medical costs [1]. Total number of people with diabetes is projected to double between 2000 and 2030 with a significantly greater rise in Asia [2, 3]. Among Asian regions, South Asia is developing as the epicentre of this escalating epidemic [4]. South Asians with diabetes have higher risk of developing cardiovascular events (CVD) [3]. This is partly due to the presence of peculiar body phenotype known as South Asian phenotype [5, 6]. It is characterized by increased waist circumference, increased waist hip ratio, excessive body fat mass, increased plasma insulin levels and insulin resistance, and an atherogenic dyslipidaemia, with low levels of HDL cholesterol and increased triglyceride levels [6]. Constellation of such various interrelated cardio metabolic risk factors is referred to as metabolic syndrome (MetS) [7]. There is strong evidence that presence of MetS increased the risk of cardiovascular mortality and morbidity [7, 8]. In addition MetS is also associated with other disorders such as thrombotic and inflammatory conditions, fatty liver disease, and reproductive disorders [1].

Although there is general agreement of the core components of the MetS such as insulin resistance/glucose intolerance, elevated blood pressure, obesity and dyslipidaemia, the diagnostic criteria put forward by various professional bodies differ based on the mandatory inclusion criteria [7]. The first formal definition of the MetS was put forward by the World Health Organization (WHO) in 1998 followed by National Cholesterol Education Program-Adult Treatment Panel III (NCEP:ATP III) and European Group for the Study of Insulin Resistance (EGIR) [7, 9]. The NCEP-ATPIII definition differs from both the WHO and the EGIR definitions as it does not recognize presence of “insulin resistance” as an mandatory criterion [10]. Differences in the diagnostic criteria of MetS led to difficulties in identifying MetS and comparing them between studies. As a solution for these controversies, International Diabetes Federation (IDF) in 2004 proposed new diagnostic criteria for MetS. The IDF diagnostic criteria included central obesity as an essential condition to make the diagnosis of the MetS [11].

Prevalence of MetS ranges from <10% to as high as 84%, depending on factors such as the population studied, ethnicity, geographical region, urban or rural environment, gender, and the definition of MetS used [12, 13]. Overall, it is estimated that 25% of the world's adult population have MetS according to IDF definition [12]. Factors such as higher socioeconomic status, physical inactivity, smoking, family history of diabetes, obesity, and sedentary lifestyle all influence the prevalence of the MetS [12]. There is accumulating evidence that MetS is more common among South Asians compared to the Caucasians [3, 14]. Previous surveys had shown that the prevalence of MetS was 34.8% in Pakistan and 25.3% in India [15]. In Sri Lanka, age-adjusted prevalence of MetS in general population was found to be 24.3% in all adults (males: 18.4%, female: 28.3%) [16].

MetS in patients with type 2 DM increases the risk of cardiovascular mortality and morbidity [17]. Thus, evaluating the MetS in individuals with type 2 DM is of importance for prevention of cardiovascular disease. It is even more important among South Asians with type 2 DM, considering their higher risk of having cardiovascular diseases. However, the prevalence of MetS among diabetes population in Sri Lanka and other South Asians countries is not sufficiently studied. Furthermore, there is no specific diagnostic criteria for MetS in patients with type 2 DM and the validity and performance of the commonly used definitions among individuals with type 2 DM is also not adequately explored. The main aim of this study was to determine the prevalence of MetS among patients with type 2DM using all three well known (WHO, IDF, and NCEP-ATP III) definitions and assess the influence of factors such as gender, glycemic control, and duration of diabetes, on the prevalence of MetS. In addition, performance of above three definitions and their concordance was also assessed.

## 2. Materials and Methods

This cross-sectional study included patients with type 2 DM who were followed up at the regional diabetes centre in Galle, Southern Sri Lanka, and this study was part of a large study on “cardiovascular risk assessment in patients with diabetes in Sri Lanka.” As described elsewhere [18], all previously diagnosed diabetes patients who are being followed up at the diabetes clinic, during the study period (January 2012 to July 2013), were eligible to participate in the study. Subjects were chosen by convenience sampling method for screening for cardio metabolic risk factors on an assigned day, with an average of 30 cases per week.

### 2.1. Inclusion Criteria

Individuals with ages ≥ 20 years and clinically determined type 2 DM were qualified to participate in the study.

### 2.2. Exclusion Criteria

Participants aged < 20 years, pregnant, and lactating mothers were excluded from the study. Also patients with other chronic illness including rheumatoid arthritis, severe osteoarthritis, symptomatic heart failure, type 1 DM, myocardial infarction within last 6 months, acromegaly, clinically apparent hypothyroidism, hypogonadism, chronic obstructive airway disease, nephrotic syndrome and chronic kidney disease (stage 3 or more), and chronic liver disease were also excluded. In addition, patients with malignancies and extreme body habitus (BMI >40), those on prolong steroid use, and those who were on active drug treatment for obesity were excluded from the study.

### 2.3. Data Collection, Anthropometric Measurements, Clinical Examination, and Laboratory Tests

A pretested interviewer-administered questionnaire was used to obtain demographic and medical information such as age, sex, ethnicity, social background, duration of diabetes, and family history of dyslipidaemia, and diabetes among first degree relatives. Anthropometric measurements including waist circumference (cm), weight (Kg), and height (m) were then measured. Waist circumference (WC) was recorded by placing a nonstretchable fibre-glass measuring tape around the waist midway between the last rib and iliac crest with the subject in the standing position. All anthropometric measurements were performed by trained nurses adhering to the WHO guidelines, using calibrated equipment. Blood pressure was measured using an electronic instrument (Omron Corporation, Tokyo, Japan), as the mean of two readings taken five minutes apart. BMI was calculated as weight (kg)/height^2^ (m^2^).

All chemical analyses were performed in the laboratory attached to the Regional Diabetic Centre mentioned above and same method of biochemical analysis was used throughout the study period. Overnight fasting venous blood samples were collected to measure HDL-C and LDL-C, serum TG, and glucose. Cholesterol esterase oxidase peroxidase-aminopyrine method was used to assess serum cholesterol and for measurement of serum TG glycerol phosphate oxidase peroxidase-aminopyrine method was used. For HDL cholesterol, direct method poly-ethylene-glycol-pretreated enzymes were used.

### 2.4. Ethical Approval

Ethical clearance for the present study was obtained under the study on “cardiovascular risk assessment in patients with type 2 diabetes in Sri Lanka,” from the Institutional Ethics Committee of the Faculty of Medicine, University of Ruhuna. Written informed consent was obtained from all study subjects in the local language.

### 2.5. Definition of the MetS

The metabolic syndrome (MetS) was defined according to the three well known (WHO, IDF, and NCEP-ATP III) definitions [7, 10].

### 2.6. IDF Definition

According to the IDF definition, ethnicity specific cut-off value for central obesity should be present as an essential criterion together with two of the following four components to diagnose MetS: raised triglycerides ≥ 150 mg/dL (1.7 mmol/L) or specific treatment for this lipid abnormality; reduced HDL cholesterol < 40 mg/dL (1.03 mmol/L) in males, < 50 mg/dL (1.29 mmol/L) in females or specific treatment for this lipid abnormality; raised blood pressure (BP): systolic BP ≥ 130 or diastolic BP ≥ 85 mm Hg or treatment of previously diagnosed hypertension and; raised fasting plasma glucose (FPG) ≥ 100 mg/dL (5.6 mmol/L), or previously diagnosed type 2 diabetes. The cut-off values of WC ≥90 cm in men or ≥80 cm in women recommended for South Asians for central obesity were used in this study [19].

### 2.7. NCEP-ATP III Definition

According to NCEP- ATP III definition, an individual can be diagnosed with MetS when any of the three following components are present: WC (>102 cm for males and >88 cm for females); plasma triglycerides (≥ 150 mg/dl); HDL cholesterol (< 40 mg/dl for males and < 50 mg/dl for females); blood pressure (≥ 130/85 mm Hg), and fasting plasma glucose (≥110 mg/dl).

### 2.8. WHO Definition

According to the WHO definition [9], MetS can be diagnosed in the presence of impaired glucose tolerance, diabetes mellitus, or insulin resistance as an essential criterion together with two or more of the following components: elevated arterial blood pressure ≥140/90 mmHg: raised triglyceride (≥150 mg/dl) or low HDL cholesterol, (<35 mg/dl for males and <39 mg/dl for females): central obesity (waist-to-hip ratio WHR: >0.90 for males and >0.85 for females), and/or BMI (>30 kg/m2): microalbuminuria (urinary albumin excretion rate ≥20 min or albumin: creatinine ratio ≥30 mg/g).

### 2.9. Statistical Analysis

All numerical data were expressed as means and standard deviations and categorical data were expressed as frequencies and proportions. Statistical significance was assumed at a p value of < 0.05. The significance of the differences between means and proportions (%) was tested using Student's* t*-test, the chi-square test or ANOVA. Univariate logistic regression analysis was used to identify the associations between individual components and the MetS according to the three definitions. Kappa (*κ*) statistics was used for finding the agreement between the three definitions. All the data were analyzed using SPSS 17.0.

## 3. Results

The baseline characteristics of the study population are summarized in Tables 1 and 2. There were 2913 type 2 DM patients, and nearly 65% of them were males. The mean age (standard deviation: SD) was 49.9 (10.2) years and females were significantly older than males (*p* = 0.03). The mean BMI (SD) was 24.5 (4.3) and 37% and 57% had global and visceral obesity, respectively.

Females had significantly higher duration of diabetes, blood pressure, total cholesterol, and LDL cholesterol. The mean values of various anthropometric, clinical, and biochemical variables measured are summarized in Tables 1 and 2 according to the gender.

**Figure 1:**
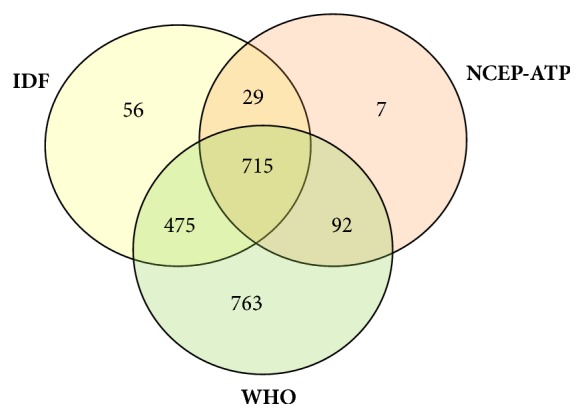
Venn diagram of metabolic syndrome according to the three different criteria.

Table 3 shows the prevalence of MetS according to the three definitions, IDF, NCEP-ATP III, and WHO. Prevalence of MetS was highest by the modified WHO definition (70%) followed by IDF (44%) and NCEP-ATP III (29%) definitions. While nearly 25% (715/2913) were identified to have MetS by all three definitions, around 45% (1311/2913) were found to have MetS by two definitions (Figure 1).

Almost all subjects recognized as having MetS by NCEP-ATP III were also identified as having MetS by one of the other two definitions. However, close to 40% (763/2055) of MetS recognized by WHO were found to have no MetS by the other two definitions. Females had higher prevalence of MetS than male according to all three definitions, and the difference was most obvious with NCEP-ATP III and IDF definitions (Table 3).

Table 4 shows the association between individual components and metabolic syndrome. According to WHO definition, presence of hypertension was associated with the highest odd of having MetS, whereas with IDF and NCEP-ATP III, low HDL was associated with the highest odds of having MetS.

As shown in Table 5, individuals with MetS according to IDF criteria had significantly higher systolic (*p* < 0.05) and diastolic blood pressure (*p* < 0.05), BMI (*p* < 0.05), and waist circumference (*p* < 0.05) compared to those with MetS based on WHO criteria. Similar observation was seen with NCEP-ATP 111 criteria as well; however, there was no significant difference of BMI and waist circumference between two groups. Individuals with MetS based on NCEP-ATP III criteria also had significantly higher BMI (*p* < 0.05), and waist circumference (*p* < 0.05), higher systolic (*p* < 0.05) and diastolic blood pressure (*p* < 0.05), and lower HDL cholesterol compared to those with MetS based on WHO. Age, duration of diabetes, and glycaemic control as indicated by HbA1c did not differ in individuals identified by the three criteria.

The agreement between the three criteria was assessed by the kappa index. The agreement between IDF with WHO and NCEP-ATP III criteria was 0.37 (*p* < 0.001) and 0.53 (*p* < 0.001), respectively, whereas the agreement between NCEP-ATP III and WHO criteria was 0.24 (*p* < 0.001).

## 4. Discussion

Even though diabetes itself increases future cardiovascular risk, recognizing MetS among patients with diabetes is vital. Accumulating research showed that the prevalence of cardiovascular diseases was significantly higher in diabetic subjects with MetS compared to those without MetS [17, 20]. Different definitions of MetS have been laid down by many different professional bodies; however, the commonly used definitions are WHO, NCEP-ATP III III, and the IDF [21, 22]. These three definitions agree on main components of MetS but differ in the cut-off values and the methods of combining the individual components. Furthermore, all three definitions were primarily used in nondiabetic individuals and, therefore, it is unclear which definition would be most suitable to recognize MetS in diabetic subjects. Overall, the prevalence of the MetS among diabetic subjects has not been adequately studied and the few available studies either had a comparatively small sample size or used only one or two definitions to assess MetS [23–26]. This is the first study to assess the prevalence of MetS among a large cohort of Sri Lankan adults with diabetes.

The most interesting finding in our study is that the prevalence of MetS varied from 28% to 70% depending on the definition used. The overall prevalence of MetS in our study was 28.9%, 43.8%, and 70.6% using NCEP-ATP III, IDF, and WHO criteria, respectively. Previous studies comparing the performance of different definitions of MetS also have shown a varied prevalence of MetS based on the definitions used [27–29]. James Osei-Yeboah et al. reported contrasting performance of the definitions with prevalence of MetS ranging from 43.83% with NCEP-ATP III, 63.58% with WHO, to 69.14% with IDF criteria [29]. In another study in India, prevalence of MetS was estimated to be 57.7% with IDF, 45.9% with NCEP-ATP III, and 28% with WHO criteria [28]. Daya Ram Pokharel et al. reported higher and contrasting prevalence of MetS among Nepalese type 2 diabetic patients (73.9% with NCEP-ATP III, 66.8% with IDF, and 69.9% with WHO) [30]. A large study involving over 4000 diabetic patients in Germany revealed comparatively lower prevalence of MetS with WHO (26.1%) and higher prevalence of MetS with IDF (82.6%) [27]. A study among diabetes patients in Cameroon revealed a comparatively higher prevalence of the MetS with IDF (71.7%) than with NCEP-ATP III (60.4%) [31]. Based on the finding of our study as well as many other studies, it is clear that the different definitions of MetS give rise to different prevalence.

The prevalence of MetS in our study was higher with the WHO definition (70.6%) than with the two other definitions; the difference undoubtedly could be attributed to the study population having diabetes and the presence of microalbuminuria as a component of MetS. In our study, the overall prevalence of microalbuminuria was as high as 45% and it may explain the higher prevalence of WHO-defined MetS. The unexpectedly high prevalence of MetS with WHO definition in our study contrasts with that reported elsewhere [27, 28]. It should be noted however that, in some of these studies, microalbuminuria was not included for analysis even though it is a part of the definition [22, 29]. However, when we look at individual components of MetS and their association with the prevalence of MetS, WHO-defined obesity (raised waist hip ratio, high BMI) appears to account for much of the higher prevalence of WHO-defined MetS in our study. The odds for the MetS in individuals with raised WHR and/or BMI were 14 times higher than the individuals with normal WHR or BMI, and this highlights the fact that raised WHR or BMI as an important predictor of MetS. Predictive ability of central obesity on the prevalence of MetS is less with other two definitions (NCEP-ATP III and IDF) and low HDL appears to be a better predictor of NCEP-ATP III and IDF defined MetS [27, 32].

Of the participants, only around 25% were recognized as having MetS simultaneously by all three definitions, suggesting that the three definitions identify a diverse group of people. Particularly this is true with WHO definition, with roughly 40% (763/2055) being classified as having MetS when other two failed to do so. In contrast, nearly all MetS recognized by the NCEP-ATP III definition were identified as having MetS by one of the other two definitions. In our study, the concordance of individuals with MetS based on IDF criteria with that of WHO and NCEP-ATP III was 0.37 and 0.53, respectively, whereas it was 0.24 between NCEP-ATP III and WHO. In contrast, previous studies revealed comparatively higher concordance between the three definitions, particularly in nondiabetic individuals. A study conducted by Deepa et al. in India had shown that the IDF had a higher agreement (0.58) with WHO definition and similar agreement with NCEP-ATP III (0.58) [22] in compared to our study. Cardiovascular Health Study (CHS) showed almost 80% concordances between NCEP-ATP III and WHO definitions [33]. Thus, the concordance between commonly used definitions of MetS seems to vary depending on factors such as ethnicity (with different cut-off values for central obesity) and presence of diabetes. It is interesting to note that despite all three criteria share most of the components, they were modest in recognizing individuals as having MetS in this study. Another noteworthy finding of our study is that the prevalence of MetS was higher among females as compared to males. Many previous studies described the similar finding of having higher prevalence of MetS in females [27, 28]. Higher prevalence of MetS in females may be due to the higher HDL cut-off and lower waist circumference cut-off values in females as compared to males. Hence, more females than males can be recognized as having central obesity or low HDL.

Identification of MetS among individuals with diabetes is important as it increase the future cardiovascular event. Bonora et al. reported fivefold increase in cardiovascular risk among individuals with type 2 diabetes having MetS compared no MetS [34]. Therefore, it is vital to recognize MetS and manage other risk factors of MetS aggressively. Our study and many other previous studies across the globe have revealed the fact that the MetS continues to be present in majority despite treatment for diabetes and other cardiovascular risk factors. This highlights the continued need for an aggressive risk factor management in individuals with diabetes [35].

As far as we are aware, our study is the first sufficiently large study to examine the prevalence of MetS in diabetic population in Sri Lanka. In addition, other strengths of the present study include its large sample size with close to 3000 individuals with diabetes and being the first ever Sri Lankan study to examine the performance of all commonly used definitions. However, there are few noteworthy limitations of our study. First, cross-sectional design of our study limits the inference of causal relationship between metabolic syndrome and cardiovascular events. Hence, performance of three definitions of MetS in our study is limited to the prevalence of MetS. Second, this study is a single centre study; therefore, generalization of our findings to the whole diabetic population in Sri Lanka may not be feasible.

In conclusion, our study demonstrates that the prevalence of metabolic syndrome among diabetic patients can significantly be varied based on the definition used. The highest prevalence of MetS was observed with WHO (70.6%) whereas lowest was observed with NCEP-ATP III definition. Raised waist circumference is an important predictor of MetS defined by all three definitions.

## Figures and Tables

**Table 1:** Baseline characteristics of study sample.

Category	Total		Male		Female		p
	Mean	SD	Mean	SD	Mean	SD	
Age	49.91	10.77	48.4	10.1	52.8	11.3	.030
Duration in years	5.04	5.71	4.8	5.8	5.4	5.6	.004
Waist circumference	90.32	10.58	90.4	10.0	89.9	11.2	.171
BMI	24.48	4.21	24.2	4.0	24.9	4.7	.060
SBP	124.30	17.40	122.5	17.4	127.5	18.2	.000
HbA1c	7.38	0.96	7.3	1.3	7.3	1.1	.464
TC	200.23	40.05	196.1	47.0	201.1	41.9	.005
LDL	125.35	36.55	122.5	39.5	126.5	37.3	.009
TG	117.15	51.20	118.6	55.9	110.3	44.6	.000
HDL	50.17	8.80	48.6	10.4	51.4	9.9	.000

**Table 2:** Baseline characteristics according to the gender.

		Male (n)	%	Female(n)	%
Age groups	20-40	548	28.5	183	28.6
	41-60	1159	60.3	641	60.4
	>60	210	10.9	172	10.9
BMI categories	<18.4	44	2.2	26	2.3
	18.5-22.9	649	33.8	316	32.5
	23-24.9	547	28.5	211	27.3
	>25	677	35.3	443	37.5
Duration of diabetes	Newly diagnosed	654	34.1	255	34.1
	<5 years	500	26.0	245	26.1
	5-10 years	362	18.8	262	18.9
	>10 years	401	23.6	234	21.0
Waist circumference	Normal	828	1.5	125	43.2
	High	1089	87.5	871	56.8
Micro-albuminuria	Present	876	45.6	440	44.1
	Absent	1041	54.4	556	55.9
Total		1917	100	996	100

**Table 3:** Prevalence of metabolic syndrome according to the three different criteria and gender.

		Total	%	Male	%	Female	%
		(n)		(n)		(n)	
IDF	No MetS	1638	56.2	1279	68	339	34
	MetS	1275	43.8	603	32	658	66
WHO	No MetS	858	29.4	618	33	230	23
	MetS	2055	70.6	1264	67	767	77
ATP 111	No MetS	2070	71.1	1594	85	456	45
	MetS	843	28.9	288	15	541	55

**Table 4:** Association between individual components and metabolic syndrome according to the three definitions.

Variables	IDF	WHO	NCEP-ATP III
	OR (95% CI)	OR (95% CI)	OR (95% CI)
High WC	2.85 (2.68- 3.03)		7.84 (6.54-9.23)
Low HDL	7.83 (6.19- 9.89)		12.81 (9.32-15.76)
TG>150	3.83 ( 3.11- 4.70)		5.26 (4.34-6.47)
HT	7.63 (6.45- 9.01)	12.46 ( 9.07-16.04)	7.31 (6.16-8.16)
High WHR or BMI		14.51 ( 13.46-16.74)	
Micro-albuminuria		6.92 ( 5.62- 8.56)	
TG>150 or HDL<35 M, <39 F		6.19 (5.89-7.88)	

**Table 5:** General characteristics of the subjects with metabolic syndrome according to the three definitions.

Variables	IDF		WHO		NCEP-ATP III	
	Mean	SD	Mean	SD	Mean	SD
Duration of diabetes in years	5.5	5.90	5.1	5.74	5.1	5.44
Age in years	47.6	78.63	46.1	62.29	47.3	68.81
Waist circumference (cm)	94.4*∗*	7.59	93.0^#^	8.89	94.4	10.09
Body mass index (kg/m^2^)	25.8*∗*	3.94	25.2^#^	4.16	26.0	4.64
Systolic blood pressure (mmHg)	131.4*∗*†	17.00	128.4^#^	17.00	133.6	16.76
Diastolic blood pressure (mmHg)	80.8*∗*†	9.63	79.7^#^	9.17	81.8	9.41
HbA_1C_ (%)	7.3	1.06	7.3	0.99	7.3	1.16
Total cholesterol (mg/dL)	201.6	42.05	200.3	40.19	201.6	42.43
LDL cholesterol (mg/dL	124.6	39.16	123.8	36.81	123.9	38.92
HDL cholesterol (mg/dL)	49.0*∗*†	9.49	49.9^#^	8.97	48.1	9.98

*∗* P< 0.05 compared to WHO criteria.

† *p* < 0.05 compared to NCEP-ATP III criteria.

^#^ *p* < 0.05 compared to NCEP-ATP III criteria.

## Data Availability

The data used to support the findings of this study are available from the corresponding author upon request.

## Abbreviations

CVD: Cardiovascular disease
MetS: Metabolic syndrome
NCEP:ATP III: National Cholesterol Education Program-Adult Treatment Panel III
WHO: World Health organization
IDF: International Diabetes Federation
DM: Diabetes mellitus
WHR: waist-to-hip ratio
WC: waist circumference.
